# Proof of Concept Trial of Dronabinol in Obstructive Sleep Apnea

**DOI:** 10.3389/fpsyt.2013.00001

**Published:** 2013-01-22

**Authors:** Bharati Prasad, Miodrag G. Radulovacki, David W. Carley

**Affiliations:** ^1^Department of Medicine, University of Illinois at ChicagoChicago, IL, USA; ^2^Department of Pharmacology, University of Illinois at ChicagoChicago, IL, USA; ^3^Department of Biobehavioral Health Science, University of Illinois at ChicagoChicago, IL, USA

**Keywords:** apnea, drug treatment, clinical trial, randomized, placebo-controlled, OSA, dronabinol

## Abstract

**Study Objective:** Animal data suggest that Δ^9^-TetraHydroCannabinol (Δ^9^THC) stabilizes autonomic output during sleep, reduces spontaneous sleep-disordered breathing, and blocks serotonin-induced exacerbation of sleep apnea. On this basis, we examined the safety, tolerability, and efficacy of dronabinol (Δ^9^THC), an exogenous Cannabinoid type 1 and type 2 (CB1 and CB2) receptor agonist in patients with Obstructive Sleep Apnea (OSA). **Design and Setting:** Proof of concept; single-center dose-escalation study of dronabinol. **Participants:** Seventeen adults with a baseline Apnea Hypopnea Index (AHI) ≥15/h. Baseline polysomnography (PSG) was performed after a 7-day washout of Continuous Positive Airway Pressure treatment. **Intervention:** Dronabinol was administered after baseline PSG, starting at 2.5 mg once daily. The dose was increased weekly, as tolerated, to 5 mg and finally to 10 mg once daily. **Measurements and Results:** Repeat PSG assessments were performed on nights 7, 14, and 21 of dronabinol treatment. Change in AHI (ΔAHI, mean ± SD) was significant from baseline to night 21 (−14.1 ± 17.5; *p* = 0.007). No degradation of sleep architecture or serious adverse events was noted. **Conclusion:** Dronabinol treatment is safe and well-tolerated in OSA patients at doses of 2.5–10 mg daily and significantly reduces AHI in the short-term. These findings should be confirmed in a larger study in order to identify sub-populations with OSA that may benefit from cannabimimetic pharmacologic therapy.

## Introduction

Pharmacologic treatment for Obstructive Sleep Apnea (OSA) is limited (Smith et al., 2006), due to the complexity of the neurochemical control and neuromodulation of central respiratory drive and the upper airway motor output (Carley and Radulovacki, 2008). Nevertheless, the poor tolerance and long-term adherence to Continuous Positive Airway Pressure (CPAP) treatment in OSA (Weaver and Grunstein, 2008), make discovery of such therapeutic alternatives clinically relevant and important.

Basic investigations support a strong neuromodulatory role of endocannabinoids on autonomic cardio-respiratory functions, which maybe distinct based on location (central vs. peripheral nervous system; Padley et al., 2003; Kopczynska, 2007), interactions with other neurotransmitters pertinent to sleep-wake behaviors (Di Marzo et al., 1998), and possibly specific receptor subtypes (Lin and Lee, 2002). The interaction of cannabinoid and serotonergic pathways is complex (Kimura et al., 1998; Cheer et al., 1999) and likely modified by similar factors. Animal data suggest that cannabinoid agonists improve respiratory stability and this effect maybe mediated by antagonism of peripheral serotonergic activity (Fan, 1995; Carley et al., 2002).

We report a translational; proof of concept study aimed to test the hypothesis that dronabinol, an exogenous CB_1_ and CB_2_ receptor agonist can reduce abnormal respiratory events and associated hypoxemia in patients with OSA. Dronabinol is FDA-approved as an antiemetic and to treat cachexia. The primary objective was to assess the safety and tolerability of dronabinol in patients with moderate to severe OSA. Therefore, by design, dose-escalation of dronabinol was permitted only if the lower dose was not associated with adverse events. Another experimental objective was to test the effect of repeated doses of dronabinol on OSA severity measured by Apnea Hypopnea Index (AHI).

## Materials and Methods

### Participants

Consenting participants (ages 21–65 years) were drawn from a single tertiary-care center referral population and underwent medical and laboratory evaluations. After a clinically adequate 7-day period of discontinuation of CPAP therapy (Kohler et al., 2011), a baseline polysomnography (PSG) was performed and participants with AHI ≥ 15 were enrolled. Exclusion criteria included: arterial oxygen saturation <75% for ≥5% of total sleep time on PSG; severe symptomatic OSA precluding withdrawal of CPAP; surgical treatment for OSA, shift work, significant weight-loss within 6 months, and any uncontrolled medical disorder. This study was approved by the IRB (UIC 2008-04294).

### Study protocol

Forty-five participants were screened; 17 were enrolled and treated with dronabinol. Participants were provided coded blister cards and instructed to self-administer one labeled pill 30 min before bedtime nightly. The treatment period comprised of dronabinol 2.5, 5.0, and 10.0 mg orally once daily for 7 days each in succession *as tolerated*. Participants maintained a sleep/drug-administration/adverse-event log daily. Follow-up PSG’s were performed on nights 7, 14, and 21. Treatment adherence was assessed by pill-count and venous blood assay of study drug at baseline and on days 7, 14, and 21. Participants returned for a final visit after 21 days for re-institution of CPAP. Adverse events were assessed by phone-calls twice-weekly and during weekly clinic visits. For 48 h prior to each PSG the participants completed a Stanford Sleepiness Scale (SSS) every 2 h while awake.

The participants were given ≥8 h time-in-bed for PSG’s with standard instrumentation for electroencephalograms (C3/A2, O2/A1), bilateral electrooculograms, electromyograms (submental, bilateral anterior tibial), electrocardiogram, oronasal airflow (thermistor and nasal pressure transducer), thoracoabdominal motions (piezo-crystal, EPM Systems), pulse oximetry, and body position. Signals were acquired and processed using ALICE5^®^ (Respironics). Study medication was administered in the laboratory on days 7, 14, and 21, 30 min before lights out.

Polysomnography’s were scored according to standard published criteria by a single blinded technologist and board-certified physician (American Sleep Disorders Association, 1992; The Report of an American Academy of Sleep Medicine Task Force, 1999). Hypopneas were scored when a reduction in airflow of >50% occurred and was associated with either an oxygen desaturation of ≥3% or an arousal.

### Statistical analysis

Safety analysis was performed on participants who received at least one dose of dronabinol. The efficacy evaluable population included all participants *completing* at least 7 days of treatment without non-compliance, including at least one on-treatment repeat PSG. Non-compliance was failure to: return for study-visits, administer or log >50% of study drug, or return study drug blistercards/unused drugs. The primary efficacy measurement was within-participant change in AHI (ΔAHI) from baseline to day 21 of treatment. Other efficacy measures included ΔAHI stratified by sleep stage, body position, first-half vs. second half of the night, and oximetry-based indices and sleep architecture. The distribution of all primary and secondary efficacy measures was checked using Anderson–Darling normality tests. The primary and secondary efficacy measures were analyzed with paired two-sided *t*-tests. The independent effects of the dose of dronabinol and baseline disease severity (AHI) on primary efficacy measure were examined with ANCOVA. The stratified analyses were defined *a priori* and no adjustment for multiple comparisons was employed for these exploratory analyses. All analyses were performed using STATA 12 and a *p*-value of <0.05 was considered significant.

## Results

### Primary endpoint: Safety

Baseline characteristics of subjects enrolled (*n* = 17) are shown in Table 1. No serious adverse events (SAEs) were reported. Dose-escalation was performed as directed by a board-certified sleep physician who performed a history and physical examination on days 8 and 15 (prior to each dose-escalation). Dose was held constant if minor adverse events or new symptoms potentially related to study drug were reported. Treatment-emergent AEs were reported in 76% of participants receiving 2.5 mg, 57% receiving 5 mg, and 75% receiving 10 mg of dronabinol. The most frequent AE was somnolence (29% at 2.5 mg, 14% at 5 mg, and 50% at 10 mg dronabinol). Increased appetite was reported in 23% of participants at 2.5 mg dronabinol and appetite stimulation was not reported with dose-escalation to 5 or 10 mg of dronabinol. No significant change in weight was noted over the 21-day treatment period in the dronabinol group (all doses; mean ± SD = −0.67 kg ± 1.91 kg; *p* = 0.24). One participant was withdrawn due to AE (palpitations) and one due to oxygen saturation falling below 75% for >5% of the total sleep time on day 7 PSG (a participant with severe OSA at baseline, AHI = 93/h).

**Table 1:** **Baseline demographic characteristics**.

Parameter	Participants (*n* = 17)
Gender (male/female)	6/11*
Age	51.6 (7.9)^#^
Race/ethnicity (W/B/H/O)	4/12/1/0*
BMI	36.1 (7.3)^#^
AHI	48.8 (24.9)^#^
Minutes SaO_2_ < 85%	11.4 (18.5)^#^
Arousal Index	47.9 (26.1)^#^
Sleep efficiency	82.6 (9.0)^#^

^#^Data presented as mean (SD); *count data; Race: W, white; B, black; H, Hispanic; O, other; SaO_2_, oxygen saturation by pulse oximetry; BMI, body mass index; AHI, apnea hypopnea index.

### Efficacy endpoints

**Figure 1:**
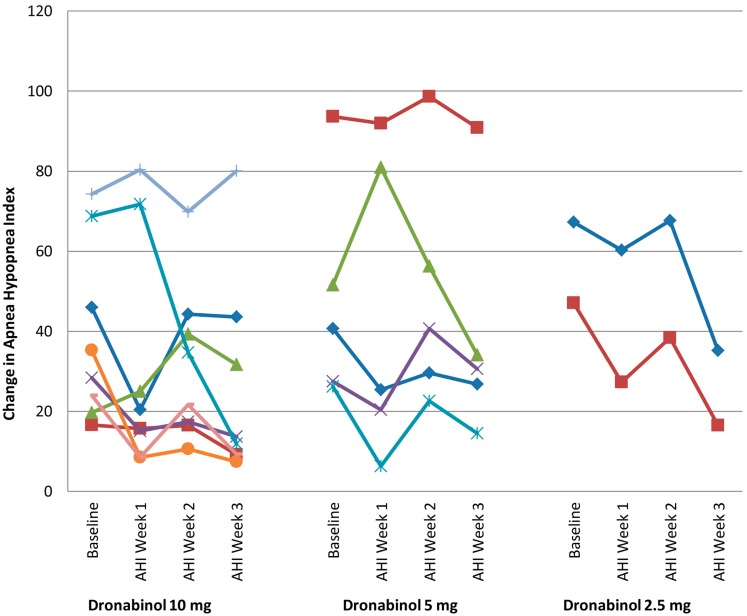
**Change in apnea hypopnea index from baseline to week 3**.

All primary and secondary efficacy measures were normally distributed with the exception of non-Supine AHI and time in minutes with <85% oxygen saturation. Table 2 presents the overall and stratified ΔAHI with 95% confidence intervals. A significant reduction in AHI from baseline (mean ± SD; −14.1 ± 17.5, *p* = 0.003) was noted *in 15 participants who completed 21 days of dronabinol treatment per protocol*. This effect was not significantly modified by dose of dronabinol (*p* = 0.84), baseline severity of OSA (baseline AHI; *p* = 0.88), or an interaction of dose and baseline AHI (*p* = 0.76). Due to the small sample size of this proof of concept study, we show the temporal change in AHI within individual participants that tolerated dronabinol at any dose for 3 weeks in Figure 1. Although, this graph confirms the overall trend toward reduction in AHI at 21 days of dronabinol treatment, there appears to be heterogeneity in treatment response in this small sample.

**Table 2:** **Primary and secondary efficacy outcomes: within-subjects change in apnea hypopnea indices with dronabinol**.

Parameter	Baseline to 3 weeks (*n* = 15) Mean (SD)	95% CI
ΔAHI	−14.11 (17.56)	−**23.8**, −**4.4**
REM ΔAHI	−5.31 (20.84)	−16.8, 6.2
NREM ΔAHI	−16.31 (18.03)	−**26.3**, −**6.3**
Supine ΔAHI	−15.23 (23.03)	−**28.5**, −**1.9**
Non-Supine ΔAHI	−9.84 (16.58)	−19.8, 0.2
ΔAHI 1st Half	−14.70 (23.79)	−**27.9**, −**1.5**
ΔAHI 2nd Half	−14.92 (18.31)	−**25.0**, −**4.8**

*ΔAHI, change in apnea hypopnea index at week 3 of dronabinol treatment compared to baseline; REM, rapid eye movement sleep; NREM, non-rapid eye movement sleep, 1st and 2nd Half, first and second 4 h of polysomnography recording time; 95% CI, Confidence Interval for paired *t*-tests*.

*Bold entries signify statistically significant changes*.

With oral dosing, the time to maximum plasma concentration of dronabinol varies from less than 30 to more than 120 min due to dronabinol’s significant first pass hepatic metabolism and high lipid solubility (initial half-life 2–6 h; Pertwee, 2009). Treatment endpoints were thus stratified according to time of night: first-half vs. second half; as well as REM vs. NREM sleep; and supine vs. non-supine sleep. While the reductions in REM AHI (−5.31 ± 20.8, *p* = 0.17) and non-supine AHI (−9.84 ± 16.6, *p* = 0.05) were not significant from baseline to end of treatment period, NREM AHI (−16.31 ± 18.0, *p* = 0.003) and supine AHI (−15.23 ± 23.0, *p* = 0.02) were reduced significantly with 3 weeks of dronabinol treatment. In addition, dronabinol treatment led to significant reductions in AHI during the first-half of the night (−14.70 ± 23.8, *p* = 0.03) as well as during the second half of the night (−14.92 ± 18.3, *p* = 0.007).

### Oximetry

Secondary endpoints to examine treatment-related improvement in oxygen delivery included change from baseline to day 21 of treatment in two measures: (1) time (minutes) spent with oxygen saturation below 85% and (2) nadir oxygen saturation. Despite improvements in AHI, dronabinol treatment did not lead to numerical improvement in nadir oxygen saturation (0.0 ± 3.0, *p* = 0.5) or any decrease in time below 85% oxygen saturation in minutes (0.9 ± 3.8, *p* = 0.4).

### Sleep architecture and sleepiness

The SSS scores at baseline were 3.44 ± 1.60 (mean ± SD), which improved to 2.99 ± 1.60 (*p* = 0.05). The dronabinol group showed no significant changes in Sleep Efficiency at week 3 compared to baseline (0.38 ± 11.3, *p* = 0.9). The Arousal Index showed no significant change at 3 weeks of dronabinol treatment compared to baseline (2.23 ± 19.2, *p* = 0.6). No significant changes were noted in the duration of REM or slow wave sleep relative to baseline.

## Discussion

This pilot study supports the safety and tolerability of dronabinol in patients with OSA. Dronabinol resulted in a significant improvement in AHI compared to baseline, with an overall reduction by 32%, a response that if replicated, may be significant in mild-moderate OSA. The therapeutic effect of dronabinol noted in this translational study is concordant with previous animal data. Pharmacologic modification of reflexes involving nodose ganglion sensory neurons can stabilize inspiratory effort (Radulovacki et al., 1998) and increase the tonic/phasic activity of the upper airway dilator muscles (Fenik et al., 2001). The vagal afferent neurons in the nodose ganglion are regulated by endocannabinoid systems. These effects vary depending on the agonist used, its receptor binding affinity, site of action, and the route of administration. For example, anandamide, an endogenous cannabinoid that binds to CB_1_ as well as vanilloid receptors evokes apnea in awake and anesthetized rats when administered intravenously (Carley et al., 2002). Another study demonstrated similar effects of HU-210 (a chemical structurally related to Δ^9^-TetraHydroCannabinol, Δ^9^THC), whereby its injection into the ventrolateral medulla of anesthetized rats abolished phrenic nerve activity, resulting in apnea (Carley et al., 2002). In contrast, Δ^9^THC, a non-selective cannabinoid receptor (CB_1_ and CB_2_) agonist administered intraperitoneally significantly alters vagal balance, stabilizes respiratory pattern, and produces a dose-dependent reduction in sleep related respiratory event density in an established rat model of sleep-disordered breathing (Carley et al., 2002). Additionally, Δ^9^THC completely blocked the apnea-genesis produced by peripheral injection of serotonin (which does not penetrate the blood brain barrier; Carley et al., 2002), suggesting that CB receptors significantly modulate serotonin signaling in the peripheral nervous system. This possibility is further supported by *in vitro* data demonstrating that activation of CB_1_ receptors on isolated nodose ganglion neurons inhibits depolarization of these neurons by serotonin (Fan, 1995). These observations provide mechanistic insights into a potentially therapeutic role for dronabinol (Δ^9^THC) in patients with OSA.

Carley et al., in their preclinical study showed that Δ^9^THC decreased respiratory rate across all sleep/wake states, with no effect on tidal volume. Thus the reduction in spontaneous and serotonin-related apnea by Δ^9^THC in this animal model was unlikely to represent a respiratory stimulant effect. Consistent with these observations, the treatment effects of dronabinol appear to be influenced by body position. We observed a significant effect in the *supine AHI* *only*, suggesting modulation of hypoglossal motor output (i.e., upper airway collapsibility) may partially account for the therapeutic effects of dronabinol. Similar significant improvements were observed with dronabinol treatment *during NREM sleep*, while no significant change in AHI was noted in REM sleep. Animal data suggest that antagonism of peripheral 5-HT_3_ serotonin receptor activity (with ondansetron) suppresses apnea and that this is a plausible pathway for similar effects noted with Δ^9^THC. These animal data disclose effects, primarily, on the expression of apnea during REM sleep. In contrast, ondansetron and dronabinol appear to have no effect on REM sleep apnea in humans. This discrepancy between animal observations and translational studies in patients with OSA, maybe due to species-specific effects or a lack of comparable doses between species. The absence of therapeutic effects of dronabinol on REM AHI presents a potential limitation to this therapeutic strategy in patients with OSA.

We did not observe concomitant improvement in oxygenation indices, likely due to the small sample size and the heterogeneity in treatment response. A subjective improvement in daytime sleepiness with dronabinol treatment was noted, suggesting a clinically significant response maybe expected in selected populations with OSA syndrome. There were no statistically significant treatment-related changes in sleep architecture compared to baseline. However, the sample showed a mean sleep efficiency of 83% at baseline, which is considered normal in the laboratory environment (possible ceiling-effect) and no significant degradation of sleep architecture was noted. No serious treatment-related adverse events were noted in this study. The majority of adverse events were reported at the 2.5 mg dose (in the first week) and improved subsequently.

### Limitations

Although dronabinol is approved for clinical use in other chronically ill populations, the tolerability of dronabinol specific to the OSA populations is unknown, where daytime somnolence, nocturnal sleep degradation, and significant weight changes for example are of particular concern. This was addressed in the protocol design of this pilot study by permitting dose-escalation as clinically tolerated, recognizing that time and dose effects maybe potentially confounded. Significant time-effects despite the short-duration of this study and in the context of a pharmacologic intervention may occur due to neuroplasticity or the achievement of steady-state drug level responses. Our repeated weekly morning serum samples for dronabinol levels were below the limit of detection (2 ng/ml) in all samples: that is daily dosing with up to 10 mg dronabinol did not result in accumulation of a steady-state plasma level of the drug over 21 days, making time-effects due to drug accumulation unlikely. The effect-size of the 10 mg dose in the fully escalated group on AHI at 3 weeks (*n* = 8), was modest compared to baseline (Cohen’s *d* = 0.56, effect-size = 0.27). Further studies are necessary to determine optimal doses and timing of administration and how these relate to sub-acute vs. chronic effects of dronabinol on the expression of sleep-disordered breathing (potential neuroplasticity). The mechanisms of the potential therapeutic effects of dronabinol noted in this proof of concept study in patients with OSA remain hypothetical. Thus, direct quantification of the relative effects of dronabinol on central respiratory rhythmogenesis vs. its influence on hypoglossal motor output in a larger study merits consideration. This will refine the target sub-populations of OSA ideally suited to this potential alternative therapeutic approach.

## Conclusion

This proof of concept study demonstrates that dronabinol is safe, well-tolerated, and reduces AHI by approximately a third over 3 weeks of oral administration. Dronabinol treatment may be a viable alternative or adjunctive therapy in selected patients with OSA. It will be important to reproduce these findings in fully powered parallel group’s studies to identify the effects of different dose-levels and duration of dronabinol treatment in patients with OSA.

## Conflict of Interest Statement

The present study was sponsored by a grant from Pier Pharmaceuticals, a for-profit business. David Carley previously served on the Board of Directors for Pier Pharmaceuticals, which has since been acquired by Cortex Pharmaceuticals.
